# Supplementary figures and images for: Fecal microbiota transplantation research output from 2004 to 2017: a bibliometric analysis
Source: PeerJ. 2019 Feb 20;7:e6411. doi: 10.7717/peerj.6411 (PMC6387576; doi:10.7717/peerj.6411)

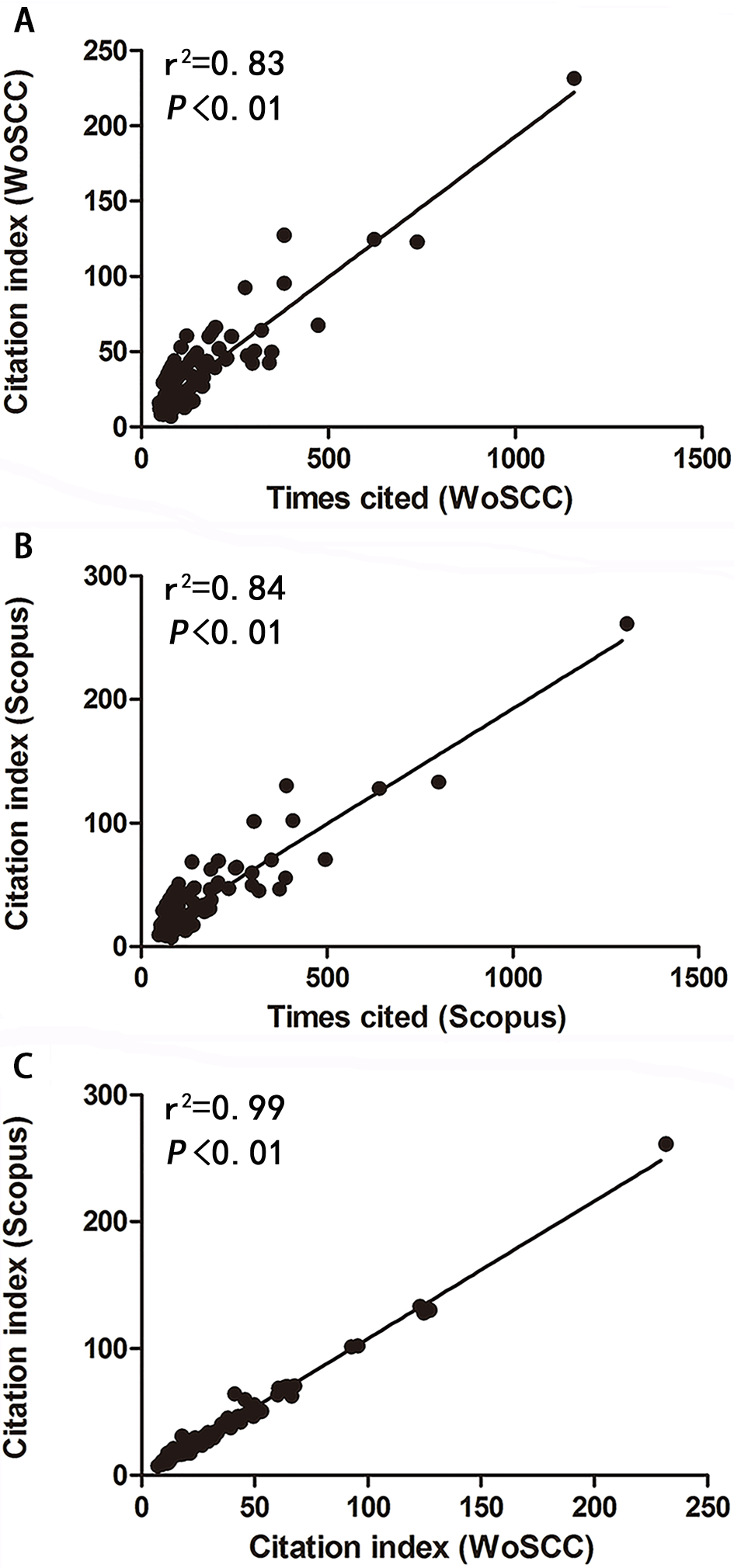

Supplement: Figure S1 [file peerj-07-6411-s001.png]

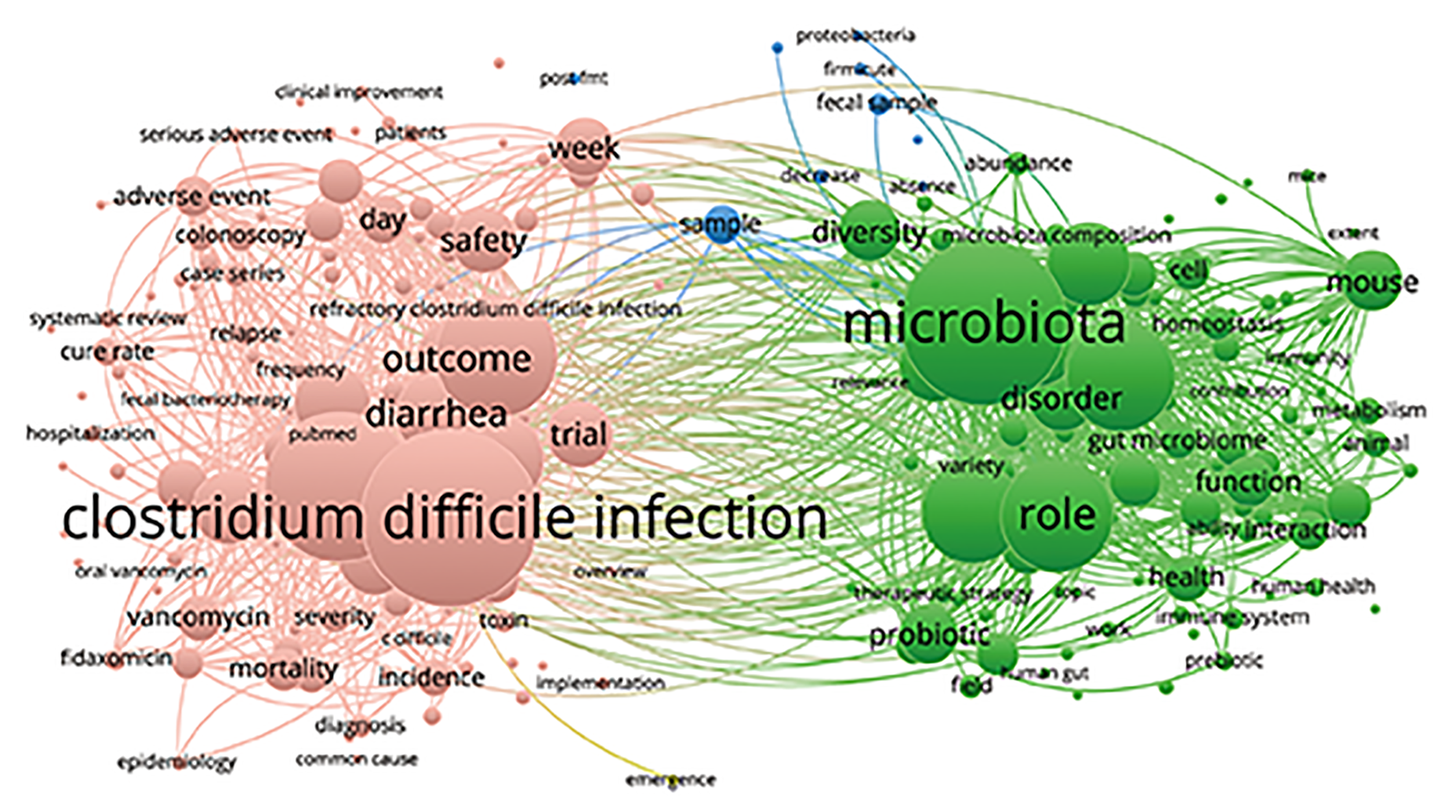

Supplement: Figure S2 [file peerj-07-6411-s002.png]
