# Supplementary material for: Fecal microbiota transplantation research output from 2004 to 2017: a bibliometric analysis
Source: PeerJ. 2019 Feb 20;7:e6411. doi: 10.7717/peerj.6411 (PMC6387576; doi:10.7717/peerj.6411)
Supplement: Table S1 [file peerj-07-6411-s003.docx]

| **Table S1. The top 100 most-cited articles ranked by the number of Times cited.** | | | | | | |
| --- | --- | --- | --- | --- | --- | --- |
| **Rank** | **Title** | **Years** | **Times cited (WoSCC)** | **Citation index (WoSCC)** | **Times cited (Scopus)** | **Citation index (Scopus)** |
| 1 | Van Nood E, Vrieze A, Nieuwdorp M, et al. Duodenal infusion of donor feces for recurrent Clostridium difficile [J]. N Engl J Med,2013,368(5):407-415. | 2013 | 1158 | 231.60 | 1307 | 261.40 |
| 2 | Vrieze A, Van N E, Holleman F, et al. Transfer of intestinal microbiota from lean donors increases insulin sensitivity in individuals with metabolic syndrome [J]. Gastroenterology, 2012, 143(4):913-916. | 2012 | 738 | 123.00 | 800 | 133.33 |
| 3 | Surawicz C M, Brandt L J, Binion D G, et al. Guidelines for diagnosis, treatment, and prevention of Clostridium difficile infections [J]. American Journal of Gastroenterology, 2013, 108(4):478-498. | 2013 | 623 | 124.60 | 641 | 128.20 |
| 4 | Gough E, Shaikh H, Manges A R. Systematic review of intestinal microbiota transplantation (fecal bacteriotherapy) for recurrent Clostridium difficile infection [J]. Clinical Infectious Diseases An Official Publication of the Infectious Diseases Society of America, 2011, 53(10):994. | 2011 | 473 | 67.57 | 495 | 70.71 |
| 5 | Vétizou M, Pitt J M, Daillère R, et al. Anticancer immunotherapy by CTLA-4 blockade relies on the gut microbiota [J]. Science, 2015, 350(6264):1079. | 2015 | 382 | 127.33 | 391 | 130.33 |
| 6 | Debast S B, Bauer M P, Kuijper E J. European Society of Clinical Microbiology and Infectious Diseases: update of the treatment guidance document for Clostridium difficile infection [J]. Clin Microbiol Infect, 2014, 20 Suppl 2:1-26. | 2014 | 382 | 95.50 | 408 | 102.00 |
| 7 | Bakken J S, Borody T, Brandt L J, et al. Treating Clostridium difficile infection with fecal microbiota transplantation. [J]. Clin Gastroenterol Hepatol, 2011, 9(12):1044-1049. | 2011 | 349 | 49.86 | 389 | 55.57 |
| 8 | Khoruts A, Dicksved J, Jansson J K, et al. Changes in the composition of the human fecal microbiome after bacteriotherapy for recurrent Clostridium difficile-associated diarrhea [J]. J Clin Gastroenterol, 2010, 44 (5):354-360. | 2010 | 342 | 42.75 | 372 | 46.50 |
| 9 | Kassam Z, Lee CH, Yuan Y, et al. Fecal microbiota transplantation for Clostridium difficile infection: systematic review and meta-analysis [J]. American Journal of Gastroenterology, 2013, 108(4):500-508. | 2013 | 321 | 64.20 | 350 | 70.00 |
| 10 | Tilg H, Kaser A. Gut microbiome, obesity, and metabolic dysfunction [J]. Journal of Clinical Investigation, 2011, 121(6):2126-2132. | 2011 | 297 | 42.43 | 317 | 45.29 |
| 11 | Brandt L J, Aroniadis O C, Mellow M, et al. Long-Term Follow-Up of Colonoscopic Fecal Microbiota Transplant for Recurrent Clostridium difficile Infection [J]. American Journal of Gastroenterology, 2012, 107(7):1079-87. | 2012 | 303 | 50.50 | 299 | 49.83 |
| 12 | Moayyedi P, Surette M G, Kim P T, et al. Fecal Microbiota Transplantation Induces Remission in Patients With Active Ulcerative Colitis in a Randomized Controlled Trial. [J]. Gastroenterology, 2015, 149(1):102-109. | 2015 | 278 | 92.67 | 304 | 101.33 |
| 13 | Hamilton M J, Weingarden A R, Sadowsky M J, et al. Standardized frozen preparation for transplantation of fecal microbiota for recurrent Clostridium difficile infection. [J]. American Journal of Gastroenterology, 2012, 107(5):761. | 2012 | 284 | 47.33 | 299 | 49.83 |
| 14 | Youngster I, Russell G H, Pindar C, et al. Oral, capsulized, frozen fecal microbiota transplantation for relapsing Clostridium difficile infection [J]. Jama, 2014, 312(17):1772-1778. | 2014 | 241 | 60.25 | 254 | 63.50 |
| 15 | Petrof E O, Gloor G B, Vanner S J, et al. Stool substitute transplant therapy for the eradication of Clostridium difficile infection: ‘RePOOPulating’ the gut [J]. Microbiome, 2013, 1(1):3. | 2013 | 229 | 45.80 | 299 | 59.80 |
| 16 | Couturier-Maillard A, Secher T, Rehman A, et al. NOD2-mediated dysbiosis predisposes mice to transmissible colitis and colorectal cancer [J]. Journal of Clinical Investigation, 2013, 123(2):700-711. | 2013 | 225 | 45.00 | 235 | 47.00 |
| 17 | Kelly C R, Ihunnah C, Fischer M, et al. Fecal microbiota transplant for treatment of Clostridium difficile infection in immunocompromised patients [J]. American Journal of Gastroenterology, 2014, 109(7):1065-1071. | 2014 | 208 | 52.00 | 207 | 51.75 |
| 18 | Norman J M, Handley S A, Baldridge M T, et al. Disease-specific Alterations in the Enteric Virome in Inflammatory Bowel Disease [J]. Cell, 2015, 160(3):447-60. | 2015 | 189 | 63.00 | 208 | 69.33 |
| 19 | Rossen N G, Fuentes S, Mj V D S, et al. Findings From a Randomized Controlled Trial of Fecal Transplantation for Patients With Ulcerative Colitis [J]. Gastroenterology, 2015, 149(1):110. | 2015 | 199 | 66.33 | 188 | 62.67 |
| 20 | Smits L P, Bouter K E C, Vos W M D, et al. Therapeutic potential of fecal microbiota transplantation. [J]. Gastroenterology, 2013, 145(5):946-53. | 2013 | 197 | 39.40 | 189 | 37.80 |
| 21 | Leffler D A, Lamont J T. Clostridium difficile infection [J]. N Engl J Med,2015,372 (16):1539-1548. | 2015 | 180 | 60.00 | NA | #VALUE! |
| 22 | Youngster I, Sauk J, Pindar C, et al. Fecal Microbiota Transplant for Relapsing Clostridium difficile Infection Using a Frozen Inoculum From Unrelated Donors: A Randomized, Open-Label, Controlled Pilot Study [J]. Clinical Infectious Diseases An Official Publication of the Infectious Diseases Society of America, 2014, 58(11):1515-22. | 2014 | 175 | 43.75 | 185 | 46.25 |
| 23 | Mattila E, Uusitalo–Seppälä R, Wuorela M, et al. Fecal Transplantation, Through Colonoscopy, Is Effective Therapy for Recurrent Clostridium difficile, Infection [J]. Gastroenterology, 2012, 142(3):490. | 2012 | 163 | 27.17 | 170 | 28.33 |
| 24 | Angelberger S, Reinisch W, Makristathis A, et al. Temporal bacterial community dynamics vary among ulcerative colitis patients after fecal microbiota transplantation [J]. American Journal of Gastroenterology, 2013, 108(10):1620-30. | 2013 | 166 | 33.20 | 168 | 33.60 |
| 25 | Colman R J, Rubin D T. Fecal microbiota transplantation as therapy for inflammatory bowel disease: A systematic review and meta-analysis [J]. Journal of Crohns & Colitis, 2014, 8(12):1569-1581. | 2014 | 164 | 41.00 | 257 | 64.25 |
| 26 | Borody T J, Khoruts A. Fecal microbiota transplantation and emerging applications. [J]. Nat Rev Gastroenterol Hepatol, 2012, 9(2):88-96. | 2012 | 164 | 27.33 | 181 | 30.17 |
| 27 | Kelly C R, Kahn S, Kashyap P, et al. Update on Fecal Microbiota Transplantation 2015: Indications, Methodologies, Mechanisms, and Outlook. [J]. Gastroenterology, 2015, 149(1):223-237. | 2015 | 148 | 49.33 | 140 | 46.67 |
| 28 | Manichanh C, Reeder J, Gibert P, et al. Reshaping the gut microbiome with bacterial transplantation and antibiotic intake. [J]. Genome Research, 2010, 20(10):1411. | 2010 | 134 | 16.75 | 138 | 17.25 |
| 29 | Bagdasarian N, Rao K, Malani P N. Diagnosis and Treatment of Clostridium difficile in Adults: A Systematic Review [J]. Jama, 2015, 313(4):398-408. | 2015 | 139 | 46.33 | 143 | 47.67 |
| 30 | Cammarota G, Ianiro G, Gasbarrini A. Fecal microbiota transplantation for the treatment of Clostridium difficile infection: a systematic review. [J]. Journal of Clinical Gastroenterology, 2014, 48(8):693. | 2014 | 135 | 33.75 | 141 | 35.25 |
| 31 | Ubeda C, Bucci V, Caballero S, et al. Intestinal Microbiota Containing Barnesiella Species Cures Vancomycin-Resistant Enterococcus faecium Colonization [J]. Infection & Immunity, 2013, 81(3):965. | 2013 | 127 | 25.40 | 144 | 28.80 |
| 32 | Kunde S, Pham A, Bonczyk S, et al. Safety, tolerability, and clinical response after fecal transplantation in children and young adults with ulcerative colitis [J]. Journal of Pediatric Gastroenterology & Nutrition, 2013, 56(6):597-601. | 2013 | 137 | 27.40 | 153 | 30.60 |
| 33 | Grehan M J, Borody T J, Leis S M, et al. Durable alteration of the colonic microbiota by the administration of donor fecal flora. [J]. Journal of Clinical Gastroenterology, 2010, 44(8):551-561. | 2010 | 139 | 17.38 | 140 | 17.50 |
| 34 | Aroniadis O C, Brandt L J. Fecal microbiota transplantation:, past, present and future. [J]. Curr Opin Gastroenterol, 2013, 29(1):79-84. | 2013 | 147 | 29.40 | 135 | 27.00 |
| 35 | Kump P K, Lackner S, Trajanoski S, et al. Alteration of intestinal dysbiosis by fecal microbiota transplantation does not induce remission in patients with chronic active ulcerative colitis. [J]. Inflammatory Bowel Diseases, 2013, 19(10):2155-65. | 2013 | 133 | 26.60 | 119 | 23.80 |
| 36 | Hartstra A V, Bouter K E, Bäckhed F, et al. Insights into the role of the microbiome in obesity and type 2 diabetes. [J]. Diabetes Care, 2015, 38(1):159-165. | 2015 | 131 | 43.67 | 126 | 42.00 |
| 37 | Levy, Maayan, Thaiss, et al. Microbiota-Modulated Metabolites Shape the Intestinal Microenvironment by Regulating NLRP6 Inflammasome Signaling [J]. Cell, 2015, 163(6):1428-1443. | 2015 | 119 | 39.67 | 121 | 40.33 |
| 38 | Lee C H, Steiner T, Petrof E O, et al. Frozen vs Fresh Fecal Microbiota Transplantation and Clinical Resolution of Diarrhea in Patients With Recurrent Clostridium difficile Infection: A Randomized Clinical Trial [J]. Jama, 2016, 315(2):142-149. | 2016 | 121 | 60.50 | 137 | 68.50 |
| 39 | Alang N, Kelly C R. Weight gain after fecal microbiota transplantation [J]. Open Forum Infect Dis, 2015, 2(1):ofv004. | 2015 | 117 | 39.00 | 129 | 43.00 |
| 40 | Floch M H, Walker W A, Madsen K, et al. Recommendations for probiotic use-2011 update [J]. J Clin Gastroenterol,2011,45 Suppl:S168-S171. | 2011 | 120 | 17.14 | 116 | 16.57 |
| 41 | Yoon S S, Brandt L J. Treatment of refractory/recurrent C. difficile-associated disease by donated stool transplanted via colonoscopy: a case series of 12 patients. [J]. Journal of Clinical Gastroenterology, 2010, 44(8):562-566. | 2010 | 110 | 13.75 | 125 | 15.63 |
| 42 | Silverman M S, Davis I, Pillai D R. Success of self-administered home fecal transplantation for chronic Clostridium difficile infection [J]. Clinical Gastroenterology & Hepatology the Official Clinical Practice Journal of the American Gastroenterological Association, 2010, 8(5):471-3. | 2010 | 111 | 13.88 | 123 | 15.38 |
| 43 | Rohlke F, Surawicz C M, Stollman N. Fecal flora reconstitution for recurrent Clostridium difficile infection: results and methodology. [J]. Journal of Clinical Gastroenterology, 2010, 44(8):567. | 2010 | 110 | 13.75 | 134 | 16.75 |
| 44 | Satokari R, Grönroos T, Laitinen K, et al. Bifidobacterium and Lactobacillus DNA in the human placenta. [J]. Letters in Applied Microbiology, 2009, 48(1):8–12. | 2009 | 115 | 12.78 | 119 | 13.22 |
| 45 | Kelly C R, De L L, Jasutkar N. Fecal microbiota transplantation for relapsing Clostridium difficile infection in 26 patients: methodology and results. [J]. Journal of Clinical Gastroenterology, 2012, 46(2):145. | 2012 | 107 | 17.83 | 185 | 30.83 |
| 46 | Drekonja D, Reich J, Gezahegn S, et al. Fecal Microbiota Transplantation for Clostridium difficile InfectionA Systematic ReviewFecal Microbiota Transplantation for Clostridium difficile Infection [J]. Annals of Internal Medicine, 2015, 313(7):725-726. | 2015 | 106 | 35.33 | 121 | 40.33 |
| 47 | Zhang F, Luo W, Shi Y, et al. Should we standardize the 1,700-year-old fecal microbiota transplantation? [J]. American Journal of Gastroenterology, 2012, 107(11):1755-6. | 2012 | 108 | 18.00 | 114 | 19.00 |
| 48 | Zheng P, Zeng B, Zhou C, et al. Gut microbiome remodeling induces depressive-like behaviors through a pathway mediated by the host's metabolism [J]. Mol Psychiatry, 2016, 21(6):786-796. | 2016 | 106 | 53.00 | 101 | 50.50 |
| 49 | Damman C J, Miller S I, Surawicz C M, et al. The microbiome and inflammatory bowel disease: is there a therapeutic role for fecal microbiota transplantation? [J]. American Journal of Gastroenterology, 2012, 107(10):1452-9. | 2012 | 103 | 17.17 | 108 | 18.00 |
| 50 | Ussar S, Griffin N, Bezy O, et al. Interactions between Gut Microbiota, Host Genetics and Diet Modulate the Predisposition to Obesity and Metabolic Syndrome [J]. Cell Metabolism, 2015, 22(3):516-530. | 2015 | 95 | 31.67 | 89 | 29.67 |
| 51 | Lo V A, Zacur G M. Clostridium difficile infection: an update on epidemiology, risk factors, and therapeutic options. [J]. Current Opinion in Gastroenterology, 2012, 28(1):1-9. | 2012 | 95 | 15.83 | 108 | 18.00 |
| 52 | De Leon L M, Watson J B, Kelly C R. Transient flare of ulcerative colitis after fecal microbiota transplantation for recurrent Clostridium difficile infection [J]. Clin Gastroenterol Hepatol,2013,11 (8):1036-1038. | 2013 | 89 | 17.80 | 98 | 19.60 |
| 53 | Weingarden A R, Chen C, Bobr A, et al. Microbiota transplantation restores normal fecal bile acid composition in recurrent Clostridium difficile infection. [J]. American Journal of Physiology Gastrointestinal & Liver Physiology, 2014, 306(4):G310. | 2014 | 90 | 22.50 | 107 | 26.75 |
| 54 | Li S S, Zhu A, Benes V, et al. Durable coexistence of donor and recipient strains after fecal microbiota transplantation [J]. Science, 2016, 352(6285):586-589. | 2016 | 88 | 44.00 | 87 | 43.50 |
| 55 | Chevalier C, Stojanović O, Colin D J, et al. Gut Microbiota Orchestrates Energy Homeostasis during Cold. [J]. Cell, 2015, 163(6):1360-74. | 2015 | 88 | 29.33 | 101 | 33.67 |
| 56 | Feng T, Qin H, Wang L, et al. Th17 Cells Induce Colitis and Promote Th1 Cell Responses through IL-17 Induction of Innate IL-12 and IL-23 Production [J]. Journal of Immunology, 2011, 186(11):6313-6318. | 2011 | 90 | 12.86 | 91 | 13.00 |
| 57 | Kelly C R, Khoruts A, Staley C, et al. Effect of Fecal Microbiota Transplantation on Recurrence in Multiply Recurrent Clostridium difficile Infection: A Randomized Trial [J]. Ann Intern Med,2016,165 (9):609-616. | 2016 | 81 | 40.50 | 86 | 43.00 |
| 58 | Mackay C, Salminen S, Wong G, et al. The gut microbiota and inflammatory noncommunicable diseases: Associations and potentials for gut microbiota therapies [J]. Journal of Allergy & Clinical Immunology, 2015, 135(1):3. | 2015 | 83 | 27.67 | 87 | 29.00 |
| 59 | Seekatz A M, Aas J, Gessert C E, et al. Recovery of the gut microbiome following fecal microbiota transplantation. [J]. Mbio, 2014, 5(3):00893-14. | 2014 | 82 | 20.50 | 93 | 23.25 |
| 60 | Brandt L J. American Journal of Gastroenterology Lecture: Intestinal Microbiota and the Role of Fecal Microbiota Transplant (FMT) in Treatment of C. difficile Infection [J]. American Journal of Gastroenterology, 2013, 108(2):177-185. | 2013 | 83 | 16.60 | 102 | 20.40 |
| 61 | Petrof E O, Khoruts A. From stool transplants to next-generation microbiota therapeutics [J]. Gastroenterology, 2014, 146(6):1573-1582. | 2014 | 82 | 20.50 | 88 | 22.00 |
| 62 | Brandt L J, Aroniadis O C. An overview of fecal microbiota transplantation: techniques, indications, and outcomes. [J]. Gastrointestinal Endoscopy, 2013, 78(2):240-9. | 2013 | 94 | 18.80 | 85 | 17.00 |
| 63 | Garborg K, Waagsbø B, Stallemo A, et al. Results of faecal donor instillation therapy for recurrent Clostridium difficile-associated diarrhoea. [J]. 2010, 42(11-12):857-861. | 2010 | 77 | 9.63 | 81 | 10.13 |
| 64 | Benakis C, Brea D, Caballero S, et al. Commensal microbiota affects ischemic stroke outcome by regulating intestinal Î³Î´ T cells [J]. Nature Medicine, 2016, 22(5):516-523. | 2016 | 77 | 38.50 | 77 | 38.50 |
| 65 | Langdon A, Crook N, Dantas G. The effects of antibiotics on the microbiome throughout development and alternative approaches for therapeutic modulation [J]. Genome Medicine, 2016, 8(1):39. | 2016 | 76 | 38.00 | 90 | 45.00 |
| 66 | Crouzet L, Gaultier E, Del'Homme C, et al. The hypersensitivity to colonic distension of IBS patients can be transferred to rats through their fecal microbiota [J]. Neurogastroenterol Motil, 2013, 25(4):e272-e282. | 2013 | 79 | 15.80 | 82 | 16.40 |
| 67 | Zipursky J S, Sidorsky T I, Freedman C A, et al. Patient attitudes toward the use of fecal microbiota transplantation in the treatment of recurrent Clostridium difficile infection [J]. Clinical Infectious Diseases An Official Publication of the Infectious Diseases Society of America, 2012, 55(12):1652. | 2012 | 75 | 12.50 | 71 | 11.83 |
| 68 | Weingarden A, González A, Vázquezbaeza Y, et al. Dynamic changes in short- and long-term bacterial composition following fecal microbiota transplantation for recurrent Clostridium difficile infection [J]. Microbiome,3,1 (2015-03-30), 2015, 3(1):10. | 2015 | 71 | 23.67 | 89 | 29.67 |
| 69 | Kassam Z, Hundal R, Marshall J K, et al. Fecal Transplant via Retention Enema for Refractory or Recurrent Clostridium difficile Infection [J]. Archives of Internal Medicine, 2012, 172(2):191-193. | 2012 | 73 | 12.17 | 76 | 12.67 |
| 70 | Rosenbaum M, Knight R, Leibel R L. The gut microbiota in human energy homeostasis and obesity. [J]. Trends in Endocrinology & Metabolism Tem, 2015, 26(9):493. | 2015 | 71 | 23.67 | 81 | 27.00 |
| 71 | Song Y, Garg S, Girotra M, et al. Microbiota Dynamics in Patients Treated with Fecal Microbiota Transplantation for Recurrent Clostridium difficile Infection [J]. Plos One, 2013, 8(11):e81330. | 2013 | 75 | 15.00 | 82 | 16.40 |
| 72 | Pang X, Hua X, Qian Y, et al. Inter-species transplantation of gut microbiota from human to pigs [J]. Isme Journal, 2007, 1(2):156-162. | 2007 | 79 | 7.18 | 81 | 7.36 |
| 73 | Schaubeck M, Clavel T, Calasan J, et al. Dysbiotic gut microbiota causes transmissible Crohn's disease-like ileitis independent of failure in antimicrobial defence [J]. Gut, 2016, 65(2):225. | 2016 | 70 | 35.00 | 75 | 37.50 |
| 74 | Rohlke F, Stollman N. Fecal microbiota transplantation in relapsing Clostridium difficile infection [J]. Therapeutic Advances in Gastroenterology, 2012, 5(6):403-20. | 2012 | 73 | 12.17 | 68 | 11.33 |
| 75 | Shanahan F, Quigley E M. Manipulation of the microbiota for treatment of IBS and IBD-challenges and controversies [J]. Gastroenterology, 2014, 146(6):1554-1563. | 2014 | 73 | 18.25 | 67 | 16.75 |
| 76 | Lemon K P, Armitage G C, Relman D A, et al. Microbiota-targeted therapies: an ecological perspective [J]. Science Translational Medicine, 2012, 4(137):137rv5. | 2012 | 70 | 11.67 | 59 | 9.83 |
| 77 | Mehal W Z. The Gordian Knot of dysbiosis, obesity and NAFLD. [J]. Nature Reviews Gastroenterology & Hepatology, 2013, 10(11):637-44. | 2013 | 71 | 14.20 | 107 | 21.40 |
| 78 | Lau K, Benitez P, Ardissone A, et al. Inhibition of type 1 diabetes correlated to a Lactobacillus johnsonii N6.2-mediated Th17 bias [J]. Journal of Immunology, 2011, 186(6):3538-3546. | 2011 | 69 | 9.86 | 73 | 10.43 |
| 79 | Konijeti G G, Sauk J, Shrime M G, et al. Cost-effectiveness of competing strategies for management of recurrent Clostridium difficile infection: a decision analysis. [J]. Clinical Infectious Diseases An Official Publication of the Infectious Diseases Society of America, 2014, 58(11):1507-14. | 2014 | 66 | 16.50 | 69 | 17.25 |
| 80 | Holmes E, Kinross J, Gibson G R, et al. Therapeutic modulation of microbiota-host metabolic interactions. [J]. Science Translational Medicine, 2012, 4(137):137rv6. | 2012 | 69 | 11.50 | 104 | 17.33 |
| 81 | Collins S M, Kassam Z, Bercik P. The adoptive transfer of behavioral phenotype via the intestinal microbiota: experimental evidence and clinical implications [J]. Curr Opin Microbiol,2013,16 (3):240-245. | 2013 | 64 | 12.80 | 65 | 13.00 |
| 82 | Russell G, Kaplan J, Ferraro M, et al. Fecal bacteriotherapy for relapsing Clostridium difficile infection in a child: a proposed treatment protocol [J]. Pediatrics, 2010, 126(1):e239. | 2010 | 70 | 8.75 | 69 | 8.63 |
| 83 | Bernstein C N. Treatment of IBD: Where We Are and Where We Are Going [J]. American Journal of Gastroenterology, 2015, 110(1):114-26. | 2015 | 63 | 21.00 | 70 | 23.33 |
| 84 | Willing B P, Vacharaksa A, Croxen M, et al. Altering Host Resistance to Infections through Microbial Transplantation [J]. Plos One, 2011, 6(10):e26988. | 2011 | 62 | 8.86 | 65 | 9.29 |
| 85 | Berg A M, Kelly C P, Francis A. Farraye MD MSc. Clostridium difficile infection in the inflammatory bowel disease patient [J]. Inflammatory Bowel Diseases, 2012, 19(1):194-204. | 2013 | 63 | 12.60 | 68 | 13.60 |
| 86 | Suskind D L, Brittnacher M J, Wahbeh G, et al. Fecal microbial transplant effect on clinical outcomes and fecal microbiome in active Crohn's disease. [J]. Inflammatory Bowel Diseases, 2015, 21(3):556-63. | 2015 | 69 | 23.00 | 67 | 22.33 |
| 87 | Khanna S, Pardi D S, Kelly C R, et al. A Novel Microbiome Therapeutic Increases Gut Microbial Diversity and Prevents Recurrent Clostridium difficile Infection. [J]. Journal of Infectious Diseases, 2016, 214(2):173-181. | 2016 | 59 | 29.50 | 58 | 29.00 |
| 88 | Baxter N T, Zackular J P, Chen G Y, et al. Structure of the gut microbiome following colonization with human feces determines colonic tumor burden [J]. Microbiome, 2014, 2(1):20. | 2014 | 63 | 15.75 | 72 | 18.00 |
| 89 | Cammarota G, Ianiro G, Cianci R, et al. The involvement of gut microbiota in inflammatory bowel disease pathogenesis: Potential for therapy [J]. Pharmacol Ther, 2015, 149:191-212. | 2015 | 65 | 21.67 | 62 | 20.67 |
| 90 | Duca F A, Sakar Y, Lepage P, et al. Replication of obesity and associated signaling pathways through transfer of microbiota from obese-prone rats [J]. Diabetes, 2014, 63(5):1624. | 2014 | 64 | 16.00 | 70 | 17.50 |
| 91 | Borody T J, Campbell J. Fecal Microbiota Transplantation: Techniques, Applications, and Issues [J]. Gastroenterol Clin North Am, 2012, 41(4):781-803. | 2012 | 63 | 10.50 | 57 | 9.50 |
| 92 | Kelly J R, Borre Y, O' B C, et al. Transferring the blues: Depression-associated gut microbiota induces neurobehavioural changes in the rat. [J]. Journal of Psychiatric Research, 2016, 82:109-118. | 2016 | 64 | 32.00 | 68 | 34.00 |
| 93 | Cui B, Feng Q, Wang H, et al. Fecal microbiota transplantation through mid-gut for refractory Crohn's disease: safety, feasibility, and efficacy trial results [J]. J Gastroenterol Hepatol, 2015, 30(1):51-58. | 2015 | 69 | 23.00 | 66 | 22.00 |
| 94 | Shahinas D, Silverman M, Sittler T, et al. Toward an Understanding of Changes in Diversity Associated with Fecal Microbiome Transplantation Based on 16S rRNA Gene Deep Sequencing [J]. Mbio, 2012, 3(5):53. | 2012 | 55 | 9.17 | 66 | 11.00 |
| 95 | Brandt L J, Reddy S S. Fecal microbiota transplantation for recurrent clostridium difficile infection [J]. Journal of Clinical Gastroenterology, 2011, 45 Suppl(Suppl):S159. | 2011 | 59 | 8.43 | 63 | 9.00 |
| 96 | Brandt L J, Reddy S S. Fecal microbiota transplantation for recurrent clostridium difficile infection [J]. Journal of Clinical Gastroenterology, 2011, 45 Suppl(Suppl):S159. | 2015 | 64 | 21.33 | 53 | 17.67 |
| 97 | Rubin T A, Gessert C E, Aas J, et al. Fecal microbiome transplantation for recurrent Clostridium difficile infection: report on a case series [J]. Anaerobe, 2013, 19(2):22-26. | 2013 | 55 | 11.00 | 48 | 9.60 |
| 98 | Neemann K, Eichele D D, Smith P W, et al. Fecal microbiota transplantation for fulminant Clostridium difficile infection in an allogeneic stem cell transplant patient. [J]. Transplant Infectious Disease, 2012, 14(6):E161–E165. | 2012 | 52 | 8.67 | 61 | 10.17 |
| 99 | Fransen F, Zagato E, Mazzini E, et al. BALB/c and C57BL/6 Mice Differ in Polyreactive IgA Abundance, which Impacts the Generation of Antigen-Specific IgA and Microbiota Diversity [J]. Immunity, 2015, 43(3):527-540. | 2015 | 48 | 16.00 | 59 | 19.67 |
| 100 | Chassaing B, Gewirtz A T. Gut microbiota, low-grade inflammation, and metabolic syndrome. [J]. Toxicologic Pathology, 2014, 42(1):49-53. | 2014 | 49 | 12.25 | 52 | 13.00 |
