# Supplementary material for: Fecal microbiota transplantation research output from 2004 to 2017: a bibliometric analysis
Source: PeerJ. 2019 Feb 20;7:e6411. doi: 10.7717/peerj.6411 (PMC6387576; doi:10.7717/peerj.6411)
Supplement: Table S2 [file peerj-07-6411-s004.docx]

| **Table S2. Major clusters of co-cited references.** | | | |
| --- | --- | --- | --- |
| **Cluster ID** | **Size** | **Silhouette** | **Mean (Year)** |
| 0 | 27 | 0.77 | 2009 |
| 1 | 26 | 0.72 | 2013 |
| 2 | 21 | 0.75 | 2007 |
| 3 | 16 | 0.87 | 2012 |
| 4 | 11 | 0.91 | 2008 |
| 5 | 6 | 0.99 | 2008 |
| 6 | 4 | 0.97 | 2008 |
