# Supplementary material for: Fecal microbiota transplantation research output from 2004 to 2017: a bibliometric analysis
Source: PeerJ. 2019 Feb 20;7:e6411. doi: 10.7717/peerj.6411 (PMC6387576; doi:10.7717/peerj.6411)
Supplement: Table S3 [file peerj-07-6411-s005.docx]

| **Table S3. The journals of the top 100 articles ranked by the number of citation.** | | | | |
| --- | --- | --- | --- | --- |
| **Rank** | **Journal** | **Country** | **No. of articles** | **IF 2017** |
| 1 | AMERICAN JOURNAL OF GASTROENTEROLOGY | USA | 10 | 10.231 |
| 2 | GASTROENTEROLOGY | USA | 8 | 20.773 |
| 3 | JOURNAL OF CLINICAL GASTROENTEROLOGY | USA | 8 | 2.968 |
| 4 | CLINICAL INFECTIOUS DISEASES | USA | 4 | 9.117 |
| 5 | CELL | USA | 3 | 31.398 |
| 6 | CLINICAL GASTROENTEROLOGY AND HEPATOLOGY | USA | 3 | 7.683 |
| 7 | INFLAMMATORY BOWEL DISEASES | USA | 3 | 4.347 |
| 8 | JAMA-JOURNAL OF THE AMERICAN MEDICAL ASSOCIATION | USA | 3 | 47.661 |
| 9 | MICROBIOME | England | 3 | 9.133 |
| 10 | NATURE REVIEWS GASTROENTEROLOGY & HEPATOLOGY | USA | 3 | 16.990 |
| 11 | ANNALS OF INTERNAL MEDICINE | USA | 2 | 19.384 |
| 12 | CURRENT OPINION IN GASTROENTEROLOGY | USA | 2 | 3.414 |
| 13 | JOURNAL OF CLINICAL INVESTIGATION | USA | 2 | 13.251 |
| 14 | JOURNAL OF IMMUNOLOGY | USA | 2 | 4.539 |
| 15 | MBIO | USA | 2 | 6.689 |
| 16 | NEW ENGLAND JOURNAL OF MEDICINE | USA | 2 | 79.258 |
| 17 | PLOS ONE | USA | 2 | 2.766 |
| 18 | SCIENCE | USA | 2 | 41.058 |
| 19 | SCIENCE TRANSLATIONAL MEDICINE | USA | 2 | 16.710 |
| 20 | WORLD JOURNAL OF GASTROENTEROLOGY | China | 2 | 3.300 |
| 21 | AMERICAN JOURNAL OF PHYSIOLOGY-GASTROINTESTINAL AND LIVER PHYSIOLOGY | USA | 1 | 3.293 |
| 22 | ANAEROBE | England | 1 | 2.742 |
| 23 | ARCHIVES OF INTERNAL MEDICINE | Poland | 1 | 2.658 |
| 24 | CELL METABOLISM | USA | 1 | 20.565 |
| 25 | CLINICAL MICROBIOLOGY AND INFECTION | England | 1 | 5.292 |
| 26 | CURRENT OPINION IN MICROBIOLOGY | England | 1 | 6.710 |
| 27 | DIABETES | USA | 1 | 7.273 |
| 28 | DIABETES CARE | USA | 1 | 13.397 |
| 29 | GASTROENTEROLOGY CLINICS OF NORTH AMERICA | USA | 1 | 3.265 |
| 30 | GASTROINTESTINAL ENDOSCOPY | USA | 1 | 7.204 |
| 31 | GENOME MEDICINE | England | 1 | 8.898 |
| 32 | GENOME RESEARCH | USA | 1 | 10.101 |
| 33 | GUT | England | 1 | 17.016 |
| 34 | INFECTION AND IMMUNITY | USA | 1 | 3.256 |
| 35 | ISME JOURNAL | USA | 1 | 9.520 |
| 36 | JOURNAL OF ALLERGY AND CLINICAL IMMUNOLOGY | USA | 1 | 13.258 |
| 37 | JOURNAL OF CROHNS & COLITIS | Netherlands | 1 | 6.637 |
| 38 | JOURNAL OF GASTROENTEROLOGY AND HEPATOLOGY | Australia | 1 | 3.483 |
| 39 | JOURNAL OF INFECTIOUS DISEASES | USA | 1 | 5.186 |
| 40 | JOURNAL OF PEDIATRIC GASTROENTEROLOGY AND NUTRITION | USA | 1 | 2.752 |
| 41 | JOURNAL OF PSYCHIATRIC RESEARCH | England | 1 | 4.000 |
| 42 | LETTERS IN APPLIED MICROBIOLOGY | England | 1 | 1.471 |
| 43 | MOLECULAR PSYCHIATRY | England | 1 | 11.640 |
| 44 | NATURE MEDICINE | USA | 1 | 32.621 |
| 45 | NEUROGASTROENTEROLOGY AND MOTILITY | England | 1 | 3.842 |
| 46 | OPEN FORUM INFECTIOUS DISEASES | USA | 1 | 3.240 |
| 47 | PEDIATRICS | USA | 1 | 5.515 |
| 48 | PHARMACOLOGY & THERAPEUTICS | England | 1 | 10.376 |
| 49 | SCANDINAVIAN JOURNAL OF INFECTIOUS DISEASES | Norway | 1 | NA |
| 50 | THERAPEUTIC ADVANCES IN GASTROENTEROLOGY | England | 1 | 4.168 |
| 51 | TRANSPLANT INFECTIOUS DISEASE | USA | 1 | 1.869 |
| 52 | TRENDS IN ENDOCRINOLOGY AND METABOLISM | USA | 1 | 10.769 |
